# Kinematic Metrics Based on the Virtual Reality System Toyra as an Assessment of the Upper Limb Rehabilitation in People with Spinal Cord Injury

**DOI:** 10.1155/2014/904985

**Published:** 2014-04-23

**Authors:** Fernando Trincado-Alonso, Iris Dimbwadyo-Terrer, Ana de los Reyes-Guzmán, Patricia López-Monteagudo, Alberto Bernal-Sahún, Ángel Gil-Agudo

**Affiliations:** ^1^Biomechanics and Technical Aids Department, National Hospital for Spinal Cord Injury, Finca la Peraleda s/n, 45071 Toledo, Spain; ^2^Indra Sistemas, Avenida de Bruselas, 33-35, Alcobendas, 28108 Madrid, Spain

## Abstract

The aim of this study was to develop new strategies based on virtual reality that can provide additional information to clinicians for the rehabilitation assessment. Virtual reality system Toyra has been used to record kinematic information of 15 patients with cervical spinal cord injury (SCI) while performing evaluation sessions using the mentioned system. Positive correlation, with a moderate and very strong association, has been found between clinical scales and kinematic data, considering only the subscales more closely related to the upper limb function. A set of metrics was defined combining these kinematic data to obtain parameters of reaching amplitude, joint amplitude, agility, accuracy, and repeatability during the evaluation sessions of the virtual reality system Toyra. Strong and moderate correlations have been also found between the metrics reaching and joint amplitude and the clinical scales.

## 1. Introduction

It has been estimated that the prevalence of spinal cord injury (SCI) is 223–755 per million inhabitants, with an incidence of 10.4–83 per million inhabitants per year [1]. Fifty percent of the patients with SCI are diagnosed as complete, and in one-third of the patients, the SCI is reported as tetraplegic.

In patients with tetraplegia, the arm and hand function is affected to a different degree, depending on the level and severity of the injury [2].

Several studies have shown that the improvement in upper extremity function is one of the greatest needs in patients with tetraplegia [3]. In this respect, therapy of the upper extremities in people with tetraplegia plays a key role during the rehabilitation.

Virtual reality (VR) has emerged in the rehabilitation context in an effort to promote task oriented and repetitive movement training of motor skills while using a variety of stimulating environments [4]. This approach can increase patient motivation, while extracting objective and accurate information enables the patient's progress to be monitored.

The aim of VR is to create a feeling of immersion within the simulated environment so that the patient's behaviour during the game resembles as much as possible the one that he would have in the real world.

There are different technologies of motion capture that permit the transfer of the actual movement of the patient to a virtual environment. One of them is the inertial measurement technology. There are several advantages of using inertial measurement systems (IMUs) as motion capture systems for VR applications, since they are compact, light, resistant to environmental interference, and easy to wear.

VR technology increases the range of possible tasks, partly automating and quantifying therapy procedures and improving patient motivation using real-time task evaluation and reward [5]. It also permits the standardization of tasks and the recording of kinematic data during the execution of these tasks, making it an interesting tool for assessment of the rehabilitation progress.

Evaluation of the SCI patient's functional status is usually carried out by means of clinical scales, although they have a high subjective component depending on the observer who scores the test. Therefore, a better understanding of human movement requires more objective testing and accurate analysis of motion to describe the arm movements more precisely and specifically during functional testing. Kinematic analysis is one such method [6].

Clinical scales are not very sensitive to slight improvements in functionality and also they are not able to establish the biomechanical characteristics that explain the clinical changes in the scores obtained by the patients during their rehabilitation. Thus, it is important to find the kinematic parameters that correlate with clinical scales. In a previous study from our group, correlations were already found between kinematic data and clinical scales [7]. These scales inform about global disability, but they include specific items related to upper limb impairment. Therefore it seems relevant to go deeper in the analysis trying to obtain a more specific correlation between kinematics and functionality.

It is important to underline that kinematic data by themselves are not always sufficiently clear and understandable for clinicians in order to reliably evaluate a patient. However, combining them to obtain new metrics could enhance their potentiality as tools for physical assessment.

The objectives of the present study are (i) to analyze the correlations between kinematic data after performing upper limb tasks included in the VR system Toyra and upper limb clinical scales; (ii) to define kinematic metrics based on data recorded by the VR system Toyra that could offer additional information to clinicians; and (iii) to analyze the correlation between the defined kinematic metrics and clinical scales by applying them to a group of 15 patients with tetraplegia.

## 2. State-of-the-Art

### 2.1. Kinematic Metrics

Quantification of upper extremity movements has been researched since many years ago. One of the first studies in this field was carried out by Fitts in 1954 with the aim of analyzing the speed-accuracy trade-off and, as a result, calculating the performance and an index of difficulty of a task from three parameters: the time spent on performing the movement, the distance, and the size of the object to be reached [8].

The interest in obtaining parameters that could provide relevant information to clinicians from the quantification of the upper extremity movements is relatively recent. To this aim, there are some studies that analyzed the movements performed by patients with neurological disorders during reaching tasks and also while drawing [9–11]. There are also studies in which a basic activity of daily living (ADL) has been analyzed, such as the drinking task, in people with stroke [12] or SCI [13].

Some of the kinematic parameters calculated to obtain information that could be clinically relevant are the time spent on the task, velocity, and range of motion during the movement [6, 13, 14]; the correlation between shoulder and elbow joint angles, that indicates the coordination during reaching tasks [11–13]; and the number of peaks in the speed profile of the hand during the movement, with lower values indicating smoother trajectories [12].

In neurological rehabilitation, the assessment of upper limb motor recovery should include smoothness, efficacy, and efficiency of the movement [10]. In this study, metrics related to these characteristics of the movement have been proposed.

- Efficacy: the percentage of the task successfully completed by patient's voluntary movement.
- Accuracy: the spatial deviation between the path followed by the patient's hand and the theoretical trajectory (in other studies it has been named “trajectory error”).
- Efficiency: it is a measure of the ratio between the length of the hand's path during the movement and the length of the theoretical trajectory.
- Smoothness: it is computed from the speed profile of the hand during the movement as the number of peaks.

These metrics are more easily applicable to reaching movements in which the theoretical hand's path is considered as the straight line between the starting point and the target location.

Most of the metrics proposed are a measure of the error or deviation between two variables. So, for example, smoothness as the number of peaks is a measure of error, since a higher number of peaks are related to a less smooth movement. The same occurs in accuracy and efficiency metrics, in which a decrease in these metrics indicates an improvement in motor performance for a functional task. For that reason, it seems necessary to obtain parameters that could be directly proportional to the patient's functional status [15].

### 2.2. Clinical Scales

There are plenty of scales in the literature which pretend to assess the patients in order to detect functional changes during the upper limb rehabilitation process [16]. These assessment scales include grasping, holding, and manipulating objects, which require the recruitment and complex integration of muscle activity from shoulder to fingers.

The upper extremity motor function tests are classified in the following categories: (1) strength tests; (2) functional tests; and (3) ADL tests [17]. In this section, only the clinical scales that were used in this study and those that will be mentioned in the “Discussion” section are described.

#### 2.2.1. Strength Tests

The evaluation of key muscle groups is important to identify the motor level in patients with tetraplegia. Motor index gives a rapid overall indication of a patient's limb impairment using principal components analysis (Hotelling's method), where the large number of movements was reduced to one movement at each joint which represented the general strength of movement at the joint [18].

#### 2.2.2. Functional Tests

Jebsen Taylor Test of Hand Function [19] is a scale which pretends to assess the hand disability and improvement in hand function gained by therapeutic procedures in patients with hand disabilities, but due to the kind of activities proposed in the test it is necessary to have a relatively high degree of dexterity to complete it.

The Action Research Arm (ARA) test provides a rapid yet reliable and standardized performance test appropriate for use in assessing recovery of upper limb but it is solely used in stroke patients.

The Fugl-Meyer Assessment (FMA) was developed to measure sensorimotor stroke recovery based on Twitchell and Brunnstrom's concept of sequential stages of motor return in patients with hemiplegic stroke [20].

The Motor Activity Log (MAL) is a scripted, structured interview that was developed by Taub et al. to measure the effects of constraint-induced movement (CI) therapy on use of the more-impaired arm outside the laboratory in individuals with stroke [21].

#### 2.2.3. ADL Tests

Two of the most used ADL evaluations for patients with tetraplegia are the functional independence measure (FIM) and the spinal cord independence measure II (SCIM II). These tests are validated and reliable and show strong correlation with each other [22].

The purpose of the FIM is the measurement of the severity of the patient's disability and the outcomes of medical rehabilitation in patients. The FIM has a good clinical interrater agreement in patients undergoing inpatient medical rehabilitation (ICC = 0.97). FIM scores were significantly lower in complete C4 tetraplegics than in C6 tetraplegics, which indicated that the FIM is sensitive enough to differentiate between different levels of injury [17].

The SCIM scale was developed specifically for SCI persons in order to make the functional assessments of persons with paraplegia or tetraplegia more sensitive to changes. The SCIM has a good interrater reliability (*r* = 0.98). Besides, the sensitivity of the SCIM is higher than the sensitivity of the FIM, showing in patients with tetraplegia that this scale missed 22% of the functional changes detected by the SCIM [17].

Regarding the kind of patients of this study, with a complete SCI at levels between C5 and C8, motor index, FIM, and SCIM tests were considered as the most suitable and, therefore, they have been chosen for this study.

## 3. New Assessment Metrics

### 3.1. Captured Raw Kinematic Data

**Figure 1:**
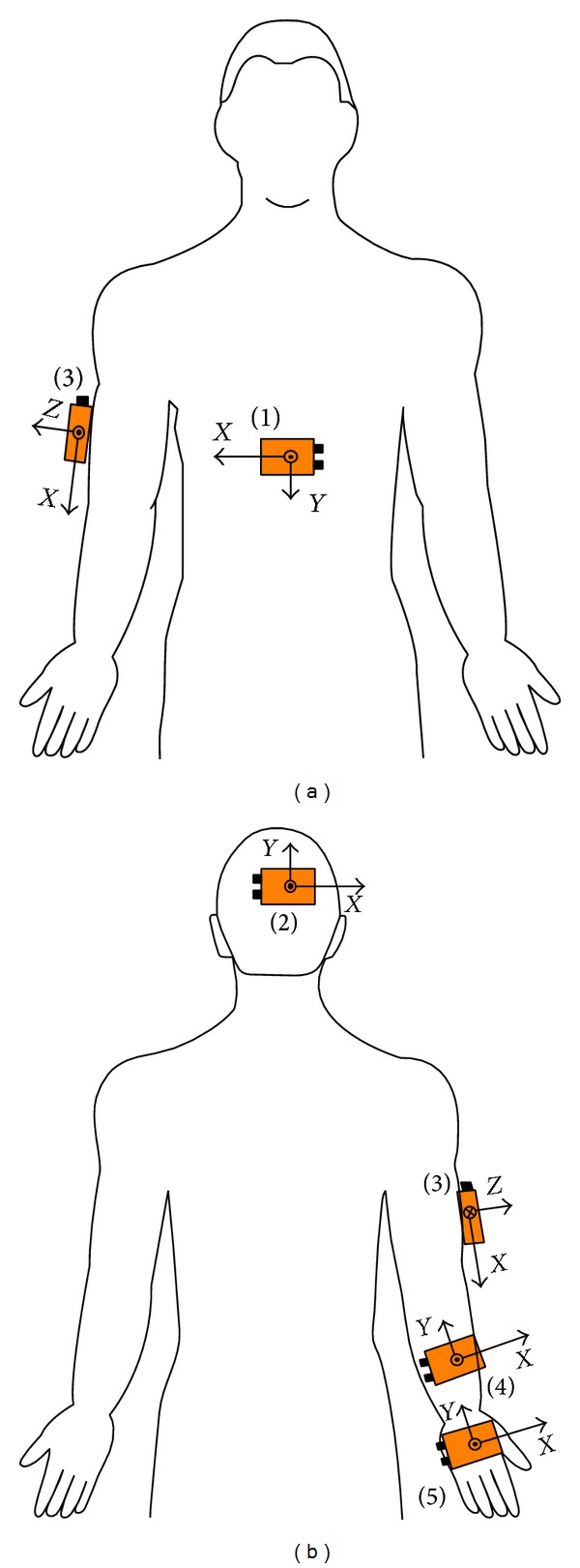
Placement of inertial sensors: (a) frontal view; (b) posterior view. The sensors were located on the trunk (1), the back of the head (2), the right arm (3), the forearm (4), and the hand (5) [23].

For the kinematic capture process, a motion capture system based on inertial sensors, MTx Xsens Company (Xsens Inc., Netherlands), has been used. In this application, 5 inertial sensors were located on the head, trunk, arm, forearm, and hand. The placement of the sensors can be seen in Figure 1.

A biomechanical model was developed, previously reported, based on inertial sensor data and upper limb (UL) anthropometric data. The MTx includes triaxis accelerometers, gyroscopes, and magnetometers. As long as the inertial sensors only provide information on the orientation of each body segment, a biomechanical model is required to calculate the angular magnitudes of clinical relevance on the basis of each orientation. The kinematic model used was based on the Euler method; thus the results depend on the sequence of rotations used. The kinematic chain proposed in this model consists of 7 DoF: three in the shoulder joint (flexion-extension, abduction-adduction, and external-internal rotation); two in the elbow joint (flexion-extension and pronation-supination); and two in the wrist (palmar-dorsal flexion and radial-ulnar deviation). More details about this biomechanical model applied here have been previously described [23].

**Figure 2:**
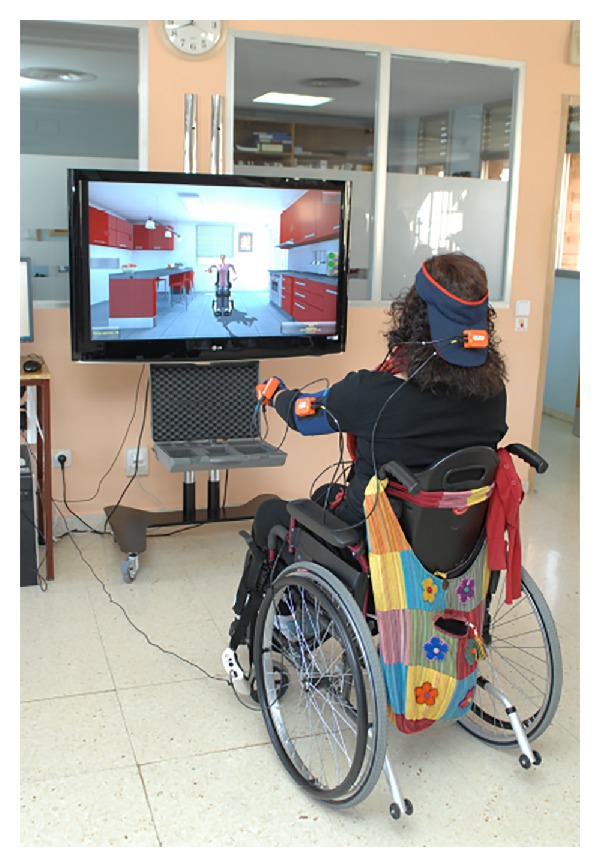
Patient performing a Toyra session.

The kinematic assessment protocol consists of the execution of an Evaluation Session with the VR System Toyra. This session comprises 14 exercises whose principal objective is to assess the patient's functional capacity, based on the record of the kinematic variables during the execution of analytical movements of the UL joints in each of its degrees of freedom. The same therapist carried out the Evaluation Sessions on all patients in order to minimize the errors due to the different placements of the sensors by different therapists. In Figure 2, the position of a patient in front of the screen during the execution of a session with Toyra can be seen.

Joint ranges of motion (ROM) of shoulder, elbow, and wrist were analysed with the mathematics software tool MATLAB (Matrix House, Cambridge, UK), thus obtaining 14 different kinematic variables: step-by-step shoulder abduction (AbdshoulderS), complete shoulder abduction (AbdshoulderC), step-by-step shoulder flexion (FlexshoulderS), complete shoulder flexion (FlexshoulderC), shoulder rotation (Rotshoulder), step-by-step elbow flexion (FlexelbowS), complete elbow flexion (FlexelbowC), elbow extension (Extelbow), elbow supination (Supelbow), elbow pronation (Proelbow), wrist extension (Extwrist), wrist flexion (Flexwrist), wrist radial deviation (Raddevwrist), and wrist ulnar deviation (Uldevwrist). The “step-by-step” variables have been measured during exercises in which the goals that the patients have to reach appear on the screen sequentially from the bottom to the top of the screen in such a way that they have to perform discrete movements and stay in the object for one second, approximately, thus requiring a certain degree of control of the muscles involved in this movement, whereas for the “complete” variables, all goals are displayed at the same time, so that the patients perform a continuous trajectory. The reason to measure separately these two kinds of variables is that “step-by-step” movements require holding the arm in a fixed position, so that the patient needs to exert the task with greater control movement. Depending on the level of SCI, some patients can be able to perform complete movements but not the step-by-step ones.

Ranges of Motion (ROM) have been calculated from the 14 kinematic variables previously mentioned as the difference between the maximum and the minimum value reached by the patients during each specific exercise.

### 3.2. New Kinematic Metrics

Five different metrics have been defined based on the kinematic data obtained during the Toyra sessions.

*(i) Joint Amplitude.* It has been defined as the sum of the ROMs obtained by a patient normalized by the corresponding ROM obtained by a healthy subject, defined as “ideal ROM”:

(1) $$\begin{array}{c} \text{JA}=\frac{\sum_{i=1}^{i=14}k_{i}\cdot {\text{ROM}}_{i}}{\sum_{i=1}^{i=14}k_{i}\cdot \text{ideal}{\text{ROM}}_{i}}\cdot 100\left[\%\right], \end{array}$$

where ROM_*i*_ (°) = degrees covered by the joint under study (it is important to remark that each exercise of the session has been designed to check the performance of a single joint. For example, the exercise of shoulder abduction will measure the shoulder's ability, despite the fact that some other joints are, to a lesser extent, also involved in this movement) and *k*_*i*_ = weighting coefficients of the exercises, chosen to give more importance to the ones that are more linked with the motor abilities of the patient.

*(ii) Reaching Amplitude.* It has been defined as the range that the patient is able to reach at the three different axes (*X*, *Y*, *Z*). The *X*-axis has been established horizontally, parallel to the screen, the *Y*-axis horizontally, perpendicular to the screen, and the *Z*-axis is vertical, parallel to the screen.

It is expected that, as long as a patient with SCI is able to reach further the objects that surround him, he would get more autonomy and functionality_._

It is calculated at each axis as the difference between the maximum and the minimum value of the position of the patient's hand, getting a range of reaching for each exercise while the patient is carrying out the three-dimensional movements required by the task. Then, these ranges of reaching are summed and normalized by the sum of ranges obtained by a healthy subject. Finally, the three values obtained for each of the three axes are weighted according to their contribution:

(2) $$\begin{array}{c} \text{RA}=\overset{j=3}{\underset{j=1}{\sum }}k_{j}\cdot \frac{\sum_{i=1}^{i=14}\max\left(h_{j_{i}}\right)-\min\left(h_{j_{i}}\right)}{\sum_{i=1}^{i=14}\max\left(\text{ideal}\;h_{j_{i}}\right)-\min\left(\text{ideal}\;h_{j_{i}}\right)}\cdot 100\left[\%\right], \end{array}$$

where *k*_*j*_ = weighting coefficient to assign to each axis a different contribution to the total reach amplitude and *h*_*j*_*i*__ = trajectory of the hand's position at each axis *j* for each exercise *i* carried out by the patient. Ideal *h*_*j*_*i*__ = trajectory of the hand's position at each axis *j* for each exercise *i* carried out by a healthy subject.

Depending on the value assigned to *k*_*j*_ (where *j* = 1 the *X*-axis, *j* = 2 the *Y*-axis, and *j* = 3 the *Z*-axis), it is possible to compute the* reaching amplitude *separately for each direction.

*(iii) Accuracy.* It has been calculated considering 2 parameters: mean distance from the trajectory performed by the patient's hand to the ideal trajectory of the hand performed by a healthy subject (*d*_mean_) and the maximum distance between these 2 trajectories (*d*_max⁡_). Consider

(3) $$\begin{array}{c} \text{Ac}=100-\overset{i=14}{\underset{i=1}{\sum }}2\cdot d_{{\text{mean}}_{i}}\cdot \left(1+\frac{d_{{\text{mean}}_{i}}}{d_{\max_{i}}}\right). \end{array}$$

The idea of this formula is to penalize the accuracy of those trajectories that present several peaks of deviation in respect to the ideal trajectory. If they have a few peaks, *d*_mean_ will not be affected to a great extent by these peaks, so that *d*_mean_ ≪ *d*_max⁡_, and thus the penalization for the accuracy would be approximately 2*d*_mean_.

However, if there are a lot of peaks of deviation, *d*_mean_ will be affected by these values, so that *d*_mean_ will increase. Consider as an example an extreme case in which there were so many peaks of deviation that *d*_mean_ ≈ *d*_max⁡_; then the penalization for the accuracy would be 4*d*_mean_, much higher than in the previous case.

In order to obtain values in percentages, as in the previous metrics, accuracy has been normalized by the value of accuracy obtained by a healthy subject:

(4) $$\begin{array}{c} {\text{Ac}}_{\mathrm{norm}}=\frac{\text{Ac}}{{\text{Ac}}_{\text{ideal}}}\cdot 100\left[\%\right]. \end{array}$$

*(iv) Agility.* It has been considered that an agile movement should not only be fast but also precise. To this aim, this metric takes into consideration three parameters: accuracy (as defined in the previous metric), angular velocity, and time needed to execute the task. Consider

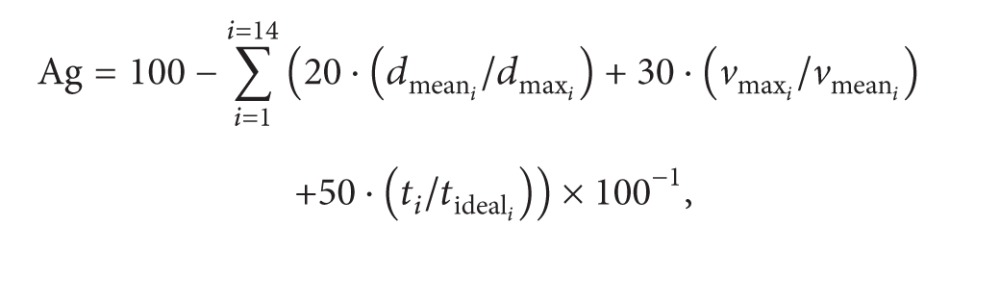
(5)
where *d*_mean_*i*__ (m) = mean distance from the trajectory performed by the patient's hand to the ideal trajectory of the hand performed by a healthy subject, *d*_max⁡_*i*__ (m) = maximum distance between the trajectory performed by the patient's hand and the ideal trajectory of the hand performed by a healthy subject, *v*_max⁡_*i*__ (°/s) = maximum angular velocity of the joint under study in each exercise, *v*_mean_*i*__ (°/s) = mean angular velocity of the joint under study in each exercise, *t*_*i*_ (s) = time spent by the patient on performing the exercise *i*, and *t*_ideal_ (s) = time spent by a healthy subject on performing the exercise *i*.

The first term of the agility penalization is the one regarding the accuracy error and it has been already explained in the previous metric.

The second term is regarding angular velocity. A very high maximum angular velocity is considered as a penalization, unless the mean velocity is also high. The reason to calculate it in this way is that patients with a badly preserved functionality will carry out the exercises quite slowly, obtaining a low mean angular velocity, but they will also carry out uncontrolled movements, for example, dropping the arm, thus getting a high maximum angular velocity. Therefore, it is important to evaluate the relationship between the maximum and the mean angular velocity, not only one of them separately.

The third term takes into account the time spent by the patient on performing the exercise in relation with the time spent by a healthy subject on performing the same exercise.

In order to obtain values in percentages, as in the previous metrics, agility has been normalized by the value of accuracy obtained by a healthy subject:

(6) $$\begin{array}{c} {\text{Ag}}_{\mathrm{norm}}=\frac{\text{Ag}}{{\text{Ag}}_{\text{ideal}}}\cdot 100\left[\%\right]. \end{array}$$

*(v) Repeatability.* It computes the inverse of the area comprised between the upper and the lower envelopes of the repetitions of the same movement during a session:

(7) $$\begin{array}{c} R=k\cdot \overset{i=8}{\underset{i=1}{\sum }}\frac{k_{0}}{A_{i}\cdot \left(1+\left(1/n_{\text{rep}}\right)\right)}, \end{array}$$

where *A*_*i*_ = area comprised between the upper and the lower envelopes of the repetitions of the exercise *I* and *k*, *k*_0_ = normalizing coefficients used to adjust the scale. Here *k* = 1000 and *k*_0_ have been used. *n*_rep_ = number of repetitions for each exercise (it is necessary that all exercises have the same number of repetitions).

**Figure 3:**
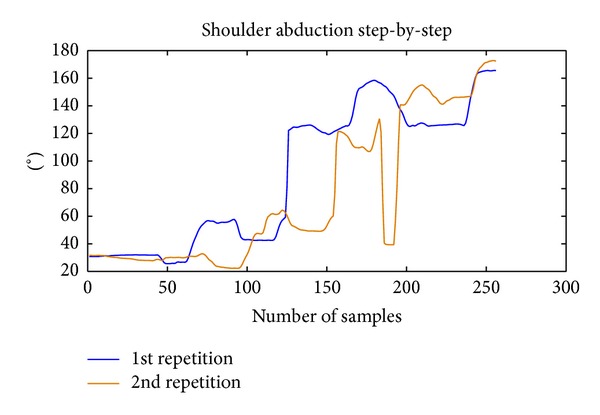
Example of the curves of shoulder abduction recorded during 2 repetitions of the same movement by a patient.

**Figure 4:**
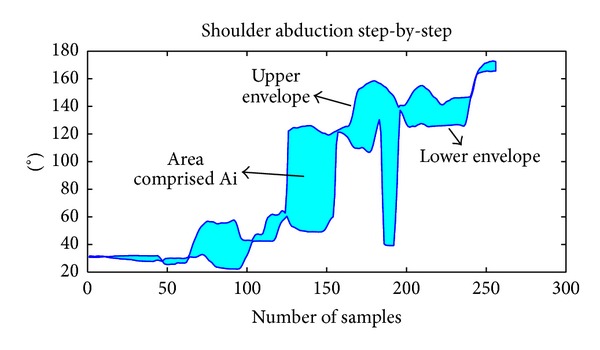
Example illustrating the calculation of the* repeatability* for the 2 repetitions previously shown in Figure 3.

For this metric, only exercises 1 to 8 have been used. They are step-by-step shoulder abduction, complete shoulder abduction, step-by-step shoulder flexion, complete shoulder flexion, step-by-step elbow flexion, complete elbow flexion, elbow extension, and shoulder rotation. These exercises are the ones that require the patient to perform a determined trajectory to accomplish the task, so the trajectories of different repetitions should be similar if the task has been correctly executed. Area *A*_*i*_ has been computed by calculating the upper and the lower envelopes along time of all repetitions of the kinematic variable corresponding to exercise *i*. For example, for the first exercise, shoulder abduction curve along time has been used, as it can be seen in Figures 3 and 4.

Area comprised in each exercise is being weighted by the number of repetitions (*n*_rep_) because the area tends to increase with the number of repetitions used.

The idea is that, as long as the patient improves his performance, he should be able to repeat more accurately the same task; thus the area between the envelopes should decrease.

## 4. Evaluation Method

### 4.1. Participants

Fifteen subjects (11 males and 4 females with complete spinal cord injury; mean age 35.33 ± 14.4 years, 4.8 ± 2.37 months since injury) participated in the study. Subject's demographic and clinical characteristics are shown in Table 1.

Eligible participants met the following criteria: (1) at least 18 years of age; (2) less than 12 months from the injury; (3) motor complete spinal cord injury according to the ASIA's impairment scale at the level of C5 to C8 (A-B ASIA level [24]); (4) no history of traumatic or cognitive pathology that can affect the Upper Limb (UL) movements; (5) normal or corrected-to-normal vision and hearing; (6) no history of technology addiction; and (7) no history of epilepsy and pregnancy. Each subject gave informed consent voluntarily which were approved by the Local Ethics Committee.

### 4.2. Data Collection and Analysis

Subjects remained seated in their own wheelchair in front of the screen. A total of five MTx IMUs were used to capture movements of the dominant UL, wirelessly connected (Bluetooth) to a computer via a digital data bus (Master Xbus), which was responsible for the synchronization, data collection, and transmission. The IMUs were strategically placed on the trunk, the back of the head, the arm, the forearm, and the hand [23]. Each subject received an explanation about how to perform the activity, which consisted of reaching the different goals that appear sequentially on the screen. Subjects were instructed to perform each of the 14 analytic movements required including complete and step-by-step shoulder, elbow, and wrist motion required. A sampling frequency of 25 Hz was used for the MTx IMUs recordings. The subjects cyclically executed each exercise three times. The mean of these three recordings yielded the final measurement value for each subject.

As it has been described in the “new kinematic metrics” section, some of the metrics require some data recorded from healthy subjects in order to compare the results of the metrics with a reference value, thus yielding a final value expressed in percentage with respect to the healthy reference. In order to obtain these reference values, a group of five healthy subjects (2 males and 3 females, mean age of 29 years and standard deviation of 6.041) was previously registered. The following parameters were extracted from the healthy subjects and then averaged to obtain the reference values: ROMs, trajectories, time spent on each exercise, and absolute value for the metrics.

Neurological examinations of all the patients were performed according to the ASIA standards [24]. The functional examination was done by using three scales. The first scale was SCIM II, which has 16 items divided into three functional areas: self-care, respiration and sphincter management, and mobility. Total score can vary from 0 (minimal) to 100 (maximal) [25]. Only the self-care subscore has been considered in this study, because it has been previously shown to be more closely related with the upper limb function [26]. From now on, this subscale will be named self-care SCIM.

The second assessment scale was the UL part of motor index scale (UL MI), which assesses power and range of the following movements: shoulder abduction, elbow flexion, and pinch between the thumb and index finger. The total score is rated between 0 (no movement) and 100 (normal movement [27]. The total score of the scale and also each of the subscores: shoulder abduction (UL MI AbdShoulder), elbow flexion (UL MI Flexelbow), and pinch (UL MI Pinch) has been evaluated.

The third scale was functional independence measure (FIM), which consists of 18 items organized in six categories, four corresponding to motor functions (self-care items, sphincter control, mobility items, and locomotion) and two corresponding to cognitive functions (communication and social cognition). The lowest and highest scores of the total ranged from 18 to 126 [28]. As in the SCIM, only the self-care subscore has been taken into account. From now on, this subscale will be named self-care FIM.

Both the kinematic assessment with Toyra and the clinical evaluation were carried out for each patient with a maximum difference of 2 days.

Descriptive analysis including means and standard deviations (SD) for continuous variables was initially performed to characterize each subject and also each group of subjects considering the neurological level of injury (C5–C8). The Pearson correlation coefficient was used to correlate kinematic ROMs with clinical and functional variables. A significance level of *P* less than 0.05 has been used. All statistical analysis was performed with Matlab (The Mathworks Inc., Natick, MA, USA).

## 5. Results

Kinematics recorded by Toyra (the 14 kinematic variables already mentioned) were obtained for each patient and averaged by levels of neurological injury. These averages can be seen in Tables 2, 3, and 4.

Values obtained by all patients in the clinical scales SCIM, UL MI, and FIM have also been obtained and averaged by level of injury, showing the results in Table 5.

Positive strong correlations between kinematic variables and clinical scales have been found in the following parameters: self-care SCIM and shoulder flexion step-by-step (*r* = 0.776, *P* = 0.00067), self-care SCIM, and complete shoulder flexion (*r* = 0.74, *P* = 0.0016), UL MI and shoulder flexion step-by-step (*r* = 0.714, *P* = 0.0028), and UL MI and complete shoulder flexion (*r* = 0.712, *P* = 0.0029).

Positive moderate correlations between kinematic variables and clinical scales have been found in the following parameters: self-care SCIM and shoulder abduction step-by-step (*r* = 0.548, *P* = 0.034), self-care SCIM and complete shoulder abduction (*r* = 0.518, *P* = 0.048), self-care SCIM and ulnar deviation (*r* = 0.551, *P* = 0.033), UL MI and shoulder abduction step-by-step (*r* = 0.547, *P* = 0.035), self-care FIM and shoulder abduction step-by-step (*r* = 0.675, *P* = 0.0113), and self-care FIM and complete shoulder flexion (*r* = 0.618, *P* = 0.0243). Results are shown in Table 6.

**Figure 5:**
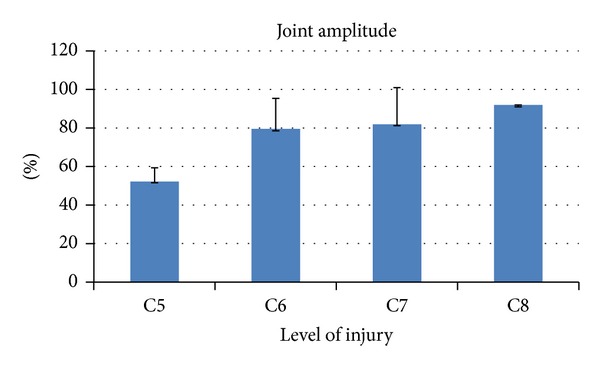
Kinematic metric* joint amplitude *per level of injury (mean ± SD). It is expressed in percentage with respect to the reference value of healthy subjects.

**Figure 6:**
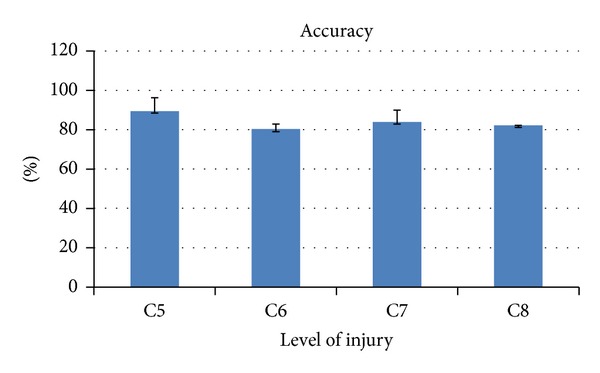
Kinematic metric* accuracy *per level of injury (mean ± SD). It is expressed in percentage with respect to the reference value of healthy subjects.

**Figure 7:**
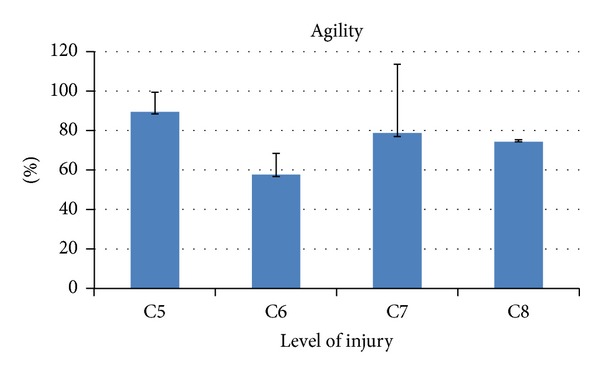
Kinematic metric* agility *per level of injury (mean ± SD). It is expressed in percentage with respect to the reference value of healthy subjects.

**Figure 8:**
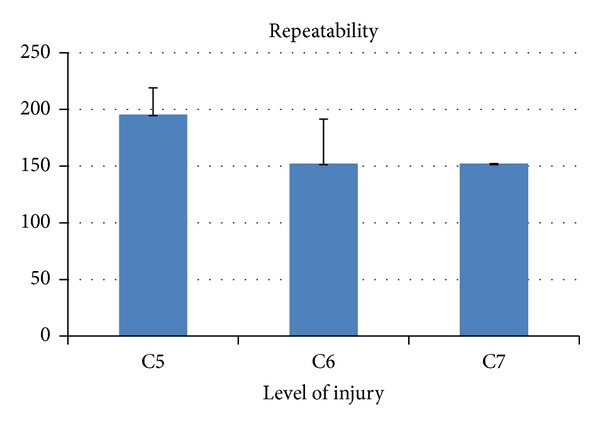
Kinematic metric* repeatability *per level of injury (mean ± SD). It is expressed in absolute value. It has been calculated only for levels C5, C6, and C7 because the number of registers for C8 level was not sufficient to establish a reliable value. For the same reason, the reference value of healthy subjects for this metric has not been calculated.

The metrics developed were applied to patients group. In Figures 5, 6, 7, and 8 the results are shown averaging the values of the metrics by levels of injury.

The metrics developed in this study have been applied to 15 patients; then the obtained values with the clinical scales' scores were compared. As it can be seen in Table 7, strong positive correlation has been found between the metric* joint amplitude *and the self-care SCIM (*r* = 0.797, *P* = 0.000375) and between this metric and the subscale UL MI AbdShoulder (*r* = 0.861, *P* = 0.00003).

There were moderate positive correlations between the following parameters:* joint amplitude *and self-care FIM (*r* = 0.591, *P* = 0.0335),* reaching amplitude (Y-axis) *and self-care FIM (*r* = 0.708, *P* = 0.00673),* reaching amplitude (Z-Axis) *and UL MI (*r* = 0.552, *P* = 0.0457),* reaching amplitude (Z-Axis) *and UL MI AbdShoulder (*r* = 0.551, *P* = 0.0332),* reaching amplitude (Z-Axis) *and UL MI Flexelbow (*r* = 0.52, *P* = 0.0467), and* reaching amplitude (Z-Axis) *and self-care FIM (*r* = 0.681, *P* = 0.01).

There was also a moderate negative correlation between* agility *and UL MI AbdShoulder (*r* = −0.536, *P* = 0.0397).

## 6. Discussion

The present study shows that the kinematic data recorded by VR system Toyra correlate with clinical scales specific for the upper limb function, which is in line with preliminary results of our group [7]. Some metrics have been defined based on these kinematic data, showing promising results in terms of clinically relevant information, as it has been demonstrated by the correlation found between some of the metrics and the self-care subscales.

This study supports the use of such VR systems not only as rehabilitation tools but also as an objective assessment tool of the user's performance, providing data with potential clinical relevance. The different degree of correlation found between the clinical scales and the kinematic variables yields interesting information that can be used in two directions. One is to analyse in minute resolution the patients' physical state, trying to use this information to complement the clinical scales scores and to design treatments that encourage the training of the joints more linked with a functional improvement. The second one would be to develop predictive models that could offer the clinician an estimation of the clinical scale score expected for a patient, thus adding objective data that could facilitate the and to follow the progression of a patient. Some previous studies go in this direction [9, 29].

The highest positive correlation between clinical scales and kinematic variables was found in the step-by-step shoulder flexion. As it was previously mentioned, the step-by-step kinematic variables require higher muscle control, and this could be the reason of the high correlation of this variable with the functionality. Together with the moderate correlations found in the shoulder abduction, these results suggest the importance of the shoulder range or movement in patients with SCI, which is consistent with previous studies that established that shoulder muscle strength, in patients with tetraplegia, is an important determinant of functional ability level [30].

In a previous study in which correlations between kinematics and clinical scales were also studied [22], no correlation was found between shoulder range of motion and any clinical scales. However, the methodology that was used in that study is quite different than the one presented here, because the patients performed only one kind of reaching and grasp task, without using any VR system, so that the reaching and grasp task did not encourage them to reach their maximum values of range of motion in all directions. In contrast with that study, here the patients carry out a wide variety of tasks, because the goals to reach are displayed in some different locations around the patient. This is one of the advantages of VR, which permits measurement of the patient's kinematics during different tasks without the difficulties of setting up a new physical environment for each task.

The only kinematic variable not related with the shoulder that showed positive correlation with clinical scales was the ulnar wrist deviation. This result could be due to the tenodesis effect, an anatomical consequence of the SCI very common in patients with level of injury C6 or C7 that entails a high wrist range of motion during the execution of the activities of daily living (ADL) [31].

Regarding the kinematic metrics developed in this study, the higher correlation obtained between the* joint amplitude *and the clinical scales, in comparison with any of the correlations obtained between the same scales and the isolated kinematic variables, suggests that the combination of kinematic variables could offer more clinically relevant information than when individually presented.

The strong positive correlation between* joint amplitude *and the SCIM scale and also the upper limb abduction shoulder subscore shows that this metric could be a good indicator of functionality. A similar result was obtained in [29], where the range of motion was found to affect to a large extent the performance of a model that predicts the clinical score from the kinematic recordings of a therapeutic robotic arm.

*Reaching amplitude *along the *Z*-axis shows moderate correlations with four of the clinical scores or subscores (UL MI global, UL MI Abdshoulder, UL MI Flexelbow, and self-care FIM scale). As it has been defined, the *Z*-axis goes vertically upwards, so that the movements in this direction require a higher force, and thus this ability could be closely related to the clinical measurements. Also* reaching amplitude *along the *Y*-axis shows a positive correlation with self-care FIM scale. The *Y*-axis was defined horizontally, perpendicular to the screen, and it is thereby the direction in which some of the ADL considered in the FIM scale take place, like eating or grooming. This could be the rationale of this correlation.

The negative correlation that showed the* agility *with the UL MI AbdShoulder was unexpected, and it could indicate that the normalization by the mean velocity used to calculate this metric could not have been enough to counteract the presence of involuntary movements, very common in patients with SCI, that usually lead to the appearance of high velocity peaks. Further filtering strategies and an optimization of the metric's parameters will be necessary to improve this metric.

With respect to the metric* accuracy*, no correlation with clinical scores was found, in contrast with a previous report, where there were strong correlations between a metric called “trajectory error,” with similar foundations to the one presented here [32]. We believe that the clinical scales (self-care SCIM, self-care FIM, and UL MI) used in our study do not encompass the specific information that this metric provides. Maybe other methods could be used in further researches to evaluate its validity. For example, in the mentioned study, clinical scales Fugl-Meyer, Motor Activity Log, Action Research Arm Test, and Jebsen-Taylor Hand Function Test were used. These scales are likely to measure aspects more related to the accuracy of movements than the ones used here.

These metrics present some limitations, such as the different number of patients in each group of injury. Therefore, it will be necessary in future researches to increase the number of patients in order to have a sufficient number to compare the averages of each level of injury. It could be also interesting to apply this metrics and kinematic analysis when the patients are performing more functional tasks such as ADLs in VR environments, not only analytical movements as in the evaluation session presented here.

## 7. Conclusions

It has been shown that some of the kinematic data extracted by a VR system based on IMUs motion capture systems have a clinical meaning. It has also been shown that the combination of these variables could provide more information than when separately used. For this purpose, a set of kinematic metrics has been defined, showing promising results in some of them. It seems very important to give clinicians the chance to obtain clinical relevant information from technological applications of rehabilitation. This could facilitate the use of such devices in clinical settings.

## Figures and Tables

**Table 1:** Demographic and clinical characteristics of the sample analysed.

Sex (male)^†^	11 (73.33)			
Age (years)*	35.33 (14.40)			
Time since injury (months)*	4.80 (2.37)			
Dominance (right)^†^	9 (60)			
ASIA (A)^†^	9 (60)			
Etiology (trauma)^†^	14 (93.33)			
Level of neurological injury (C5–C8)^†^	C5 = 7 (46.66)	C6 = 4 (26.66)	C7 = 3 (20)	C8 = 1 (6.66)

*Continuous variables are expressed as mean and standard deviation values. ^†^Categorical variables are expressed as frequency and percentage of the sample analyzed.

**Table 2:** Shoulder kinematics per level of injury (mean ± SD).

	AbdshoulderS	AbdshoulderC	FshoulderS	FshoulderC	Rotshoulder
C5 *n* = 7	73.184 ± 28.436	72.402 ± 36.022	103.506 ± 53.465	107.957 ± 41.308	114.707 ± 31.245

C6 *n* = 4	95.903 ± 34.925	122.465 ± 26.207	157.989 ± 28.381	138.222 ± 56.126	89.824 ± 22.948

C7 *n* = 3	102.218 ± 52.31	113.985 ± 45.117	165.138 ± 32.002	152.904 ± 21.112	108.454 ± 47.901

C8 *n* = 1	137.787 ± 12.10	152.151 ± 13.21	178.582 ± 12.34	175.32 ± 14.25	130.843 ± 12.120

**Table 3:** Elbow kinematics per level of injury (mean ± SD).

	FelbowC	Extelbow	FelbowS	Supelbow	Proelbow
C5 *n* = 7	118.624 ± 15.864	126.714 ± 19.974	111.632 ± 27.046	162.411 ± 85.775	146.391 ± 17.788

C6 *n* = 4	129.835 ± 10.935	145.311 ± 25.908	125.537 ± 22.501	126.215 ± 9.024	185.726 ± 58.672

C7 *n* = 3	132.846 ± 6.68	145.044 ± 9.539	131.95 ± 2.635	142.297 ± 31.714	178.916 ± 39.569

C8 *n* = 1	112.46 ± 13.23	151.505 ± 32.12	116.905 ± 12.23	122.997 ± 24.12	183.384 ± 21.14

**Table 4:** Wrist kinematics per level of injury (mean ± SD).

	Extwrist	Flexwrist	Raddevwrist	Uldevwrist
C5 *n* = 7	57.204 ± 11.602	44.053 ± 17.086	24.878 ± 10.11	23.155 ± 11.656

C6 *n* = 4	44.275 ± 21.867	47.589 ± 13.546	20.796 ± 8.173	25.851 ± 15.579

C7 *n* = 3	77.045 ± 9.831	65.793 ± 8.925	36.476 ± 2.415	42.669 ± 1.238

C8 *n* = 1	56.002 ± 12.02	54.004 ± 11.23	23.656 ± 11.21	34.868 ± 10.25

**Table 5:** Clinical subscales of self-care SCIM, UL MI, and self-care FIM per level of injury (mean ± SD).

	Self-care SCIM	UL MI	Self-care FIM
C5 *n* = 7	2 ± 1.414	66.429 ± 20.999	10 ± 2.828

C6 *n* = 4	3 ± 1.414	64.25 ± 17.115	13 ± 9.539

C7 *n* = 3	5 ± 1.732	69 ± 19.079	12 ± 2

C8 *n* = 1	8 ± 0	93 ± 0	16 ± 0

**Table 6:** Correlations found between kinematic variables recorded by VR system Toyra and clinical subscales.

	Self-care SCIM	UL MI	Self-care FIM
AbdshoulderS	*r* = 0.548*	*r* = 0.547*	*r* = 0.675*
	*P* = 0.034	*P* = 0.035	*P* = 0.0113

AbdshoulderC	*r* = 0.518*	*r* = 0.385	*r* = 0.551
	*P* = 0.048	*P* = 0.157	*P* = 0.074

FshoulderS	*r* = 0.776***	*r* = 0.714**	*r* = 0.476
	*P* = 0.00067	*P* = 0.0028	*P* = 0.1

FshoulderC	*r* = 0.74**	*r* = 0.712**	*r* = 0.618*
	*P* = 0.0016	*P* = 0.0029	*P* = 0.0243

Udwrist	*r* = 0.551*	*r* = 0.336	*r* = 0.165
	*P* = 0.033	*P* = 0.221	*P* = 0.59

*P < 0.05.

**P < 0.01.

***P < 0.001.

**Table 7:** Correlations between kinematic metrics and clinical subscales.

	Self-care SCIM	UL MI	UL MI AbdShoulder	UL MI Flexelbow	UL MI Pinch	Self-care FIM
Joint amplitude	*r* = 0.797***	*r* = 0.513	*r* = 0.861***	*r* = 0.292	*r* = 0.276	*r* = 0.591*
	*P* = 0.000375	*P* = 0.05	*P* = 0.00003	*P* = 0.291	*P* = 0.32	*P* = 0.0335

Reaching amplitude (total)	*r* = −0.068	*r* = 0.376	*r* = −0.041	*r* = −0.024	*r* = 0.346	*r* = 0.539
	*P* = 0.811	*P* = 0.167	*P* = 0.883	*P* = 0.931	*P* = 0.207	*P* = 0.057

Reaching amplitude (*X*-axis)	*r* = −0.374	*r* = 0.05	*r* = −0.393	*r* = −0.23	*r* = 0.14	*r* = 0.019
	*P* = 0.17	*P* = 0.858	*P* = 0.147	*P* = 0.409	*P* = 0.0619	*P* = 0.952

Reaching amplitude (*Y*-axis)	*r* = 0.217	*r* = 0.4	*r* = 0.258	*r* = 0.005	*r* = 0.315	*r* = 0.708**
	*P* = 0.17	*P* = 0.139	*P* = 0.354	*P* = 0.986	*P* = 0.252	*P* = 0.0067

Reaching amplitude (*Z*-axis)	*r* = 0.474	*r* = 0.523*	*r* = 0.551*	*r* = 0.52*	*r* = 0.315	*r* = 0.681*
	*P* = 0.075	*P* = 0.0457	*P* = 0.0332	*P* = 0.0467	*P* = 0.252	*P* = 0.01

Accuracy	*r* = −0.239	*r* = −0.174	*r* = −0.364	*r* = −0.442	*r* = −0.062	*r* = −0.283
	*P* = 0.391	*P* = 0.535	*P* = 0.182	*P* = 0.099	*P* = 0.828	*P* = 0.349

Agility	*r* = −0.259	*r* = −0.248	*r* = −0.536*	*r* = −0.463	*r* = −0.081	*r* = −0.338
	*P* = 0.351	*P* = 0.373	*P* = 0.0397	*P* = 0.082	*P* = 0.775	*P* = 0.26

*P < 0.05.

**P < 0.01.

***P < 0.001.
